# Chemoradiotherapy With or Without Simultaneous Integrated Boost for Cervical Cancer With Full-Thickness Stromal Invasion: A Phase 3 Randomized Clinical Trial

**DOI:** 10.1001/jamanetworkopen.2025.32501

**Published:** 2025-09-19

**Authors:** Suping Liu, Chi Fang, Xiaohua Wu, Jun Zhu, Guihao Ke

**Affiliations:** 1 Department of Gynecologic Oncology, Fudan University Shanghai Cancer Center, Shanghai, China; 2 Department of Oncology, Shanghai Medical College, Fudan University, Shanghai, China

## Abstract

**Importance:**

Patients with cervical cancer exhibiting full-thickness (FT) and outer full-thickness (OFT) stromal invasion after radical hysterectomy face high risks of recurrence, yet optimal adjuvant radiotherapy strategies remain debated.

**Objective:**

To determine if simultaneous integrated boost (SIB) radiotherapy increases 3-year progression-free survival (PFS) by 13% vs conventional chemoradiotherapy (CRT) among patients with cervical cancer exhibiting FT or OFT stromal invasion after surgery.

**Design, Setting, and Participants:**

This phase 3, single-center, randomized clinical superiority trial enrolled 466 patients in Shanghai Cancer Center with FIGO (International Federation of Gynecology and Obstetrics) 2018 stage IB to IIA and IIIC cervical cancer and pathologically confirmed FT or OFT stromal invasion from October 15, 2019, to September 20, 2024. Data were analyzed in October 2024.

**Interventions:**

Patients were randomized to receive conventional CRT (50.4 Gy in 28 fractions plus cisplatin, 40 mg/m^2^/wk) or CRT plus SIB radiotherapy (58.8 Gy in 28 fractions).

**Main Outcomes and Measures:**

The primary end point was 3-year PFS. Secondary end points included overall survival (OS), acute and late toxic effects, and progression patterns. All outcomes were analyzed under the intention-to-treat principle.

**Results:**

A total of 466 patients with cervical cancer presenting with FT or OFT stromal invasion after radical hysterectomy were randomized (233 in non-SIB radiotherapy group: median age, 53 years [IQR, 46-59 years]; 233 in SIB radiotherapy group: median age, 55 years [IQR, 48-60 years]). At a median follow-up of 33 months (range, 8-54 months), SIB radiotherapy significantly improved 3-year PFS compared with conventional CRT (84.6% vs 76.8%; *P* = .04), corresponding to a 35.7% reduction in progression risk (hazard ratio [HR], 0.64; 95% CI, 0.42-0.99; *P* = .04). Multivariable analysis identified adenocarcinoma (HR, 2.68; 95% CI, 1.62-4.44; *P* < .001), adenosquamous carcinoma (HR, 2.66; 95% CI, 1.06-6.66; *P* = .04), tumor size of 4 cm or more (HR, 1.81; 95% CI, 1.17-2.81; *P* = .01), lymphovascular space invasion (HR, 2.88; 95% CI, 1.45-5.72; *P* = .003), and positive vaginal margins (HR, 4.60; 95% CI, 1.97-10.73; *P* < .001) as independent risk factors of poorer PFS, while SIB radiotherapy remained protective (HR, 0.59; 95% CI, 0.38-0.91; *P* = .02). Profiles of toxic effects were comparable between groups.

**Conclusions and Relevance:**

In this randomized clinical trial of postoperative patients with cervical cancer presenting with FT or OFT stromal invasion, SIB radiotherapy significantly enhanced PFS without increased toxic effects. The clinical benefits and comparable profiles of toxic effects support its integration into adjuvant care.

**Trial Registration:**

Chinese Clinical Trial Registry Identifier: ChiCTR1900027272

## Key Points

**Question:**

Does simultaneous integrated boost (SIB) radiotherapy improve 3-year progression-free survival (PFS) among patients with postoperative cervical cancer with full-thickness and outer full-thickness stromal invasion?

**Findings:**

In this phase 3 randomized clinical trial of 466 patients, SIB radiotherapy was associated with a statistically significant improvement in 3-year PFS, with an absolute increase of 7.8% (84.6% vs 76.8%).

**Meaning:**

This study suggests that SIB radiotherapy may improve overall disease control for patients in this cohort, supporting its integration into adjuvant therapy.

## Teaser

This randomized clinical trial examines if simultaneous integrated boost radiotherapy increases 3-year progression-free survival vs conventional chemoradiotherapy among patients with cervical cancer exhibiting full-thickness or outer full-thickness stromal invasion after surgery.

## Introduction

Cervical cancer continues to impose a substantial global health burden, with an estimated 660 000 new cases and 350 000 deaths reported worldwide in 2022, maintaining its position as the fourth most prevalent malignant neoplasm among women.^1^ Although developed regions have witnessed a more than 50% reduction in the incidence of cervical cancer since the 1970s through cytologic screening and human papillomavirus vaccination programs,^2^ this progress remains unequally distributed. Developing regions—particularly sub-Saharan Africa, South America, Southeast Asia, and China—bear disproportionate burdens, with China alone accounting for 18% of global cases and 17% of cervical cancer mortality.^3^ Compounding this disparity, China documented a 2.7% annual increase in cervical cancer mortality across all age groups from 2005 to 2020, reaching 44 750 deaths in 2020,^4^ alongside emerging epidemiologic patterns showing regional variations, urban-rural divides, and earlier disease onset.^5,6^

Current therapeutic strategies, while generally aligned with National Comprehensive Cancer Network (NCCN) guidelines, face ongoing debates regarding optimal management of specific stages (IB3, IIA2, and IIIC). In China’s clinical landscape, radical hysterectomy followed by adjuvant chemoradiotherapy (CRT) constitutes the predominant therapeutic paradigm for cervical cancer, a treatment algorithm shaped primarily by constrained health care resources and sociodemographic factors influencing patient decision-making. This paradigm frequently uncovers complex pathologic features, particularly full-thickness (FT) and outer full-thickness (OFT) stromal invasion, defined at our institution as complete stromal involvement without parametrial transition zone extension (FT stromal invasion) vs microscopic transition zone involvement without macroscopic parametrial spread (OFT stromal invasion) (eTable 1 and eFigure 1 in Supplement 2). Although current Sedlis criteria are used to guide in the management of intermediate-risk cases based on stromal invasion depth,^7^ critical knowledge gaps persist: whether FT and OFT cases warrant distinct therapeutic approaches compared with deep stromal invasion, and whether their prognostic trajectories truly parallel conventional classifications.

A retrospective analysis of 3298 surgically treated patients (2006-2014) with two-thirds or more stromal invasion depth revealed prognostic stratification; 5-year disease-free survival (DFS) rates decreased from 85.6% for deep stromal invasion to 75.8% of FT cases, and 57.8% of OFT cases (*P* < .001), with 5-year overall survival (OS) rates of 89.9% for deep stromal invasion, 79.5% of FT cases, and 60.2% of OFT cases. Pelvic recurrence rates further highlighted this gradient (8.6% of FT cases vs 17.0% of OFT cases). Subgroup analysis of 217 FT cases identified postoperative radiotherapy as an independent prognostic determinant for both DFS and OS.^8^ Anatomical investigations elucidated potential mechanisms; FT and OFT invasion frequently involve microscopic dissemination to pelvic structures (bladder posterior wall, uterosacral and main ligaments), correlating with observed recurrence patterns.

This anatomical precision informed our development of simultaneous integrated boost (SIB) radiotherapy (58.8 Gy/28 fractions, equivalent dose delivered in 2 Gy [EQD2], 59.29 Gy) targeting subclinical lesions. Unlike sequential techniques requiring extended treatment courses, SIB radiotherapy achieves dose escalation within standard treatment time through optimized dose gradients, improving nodal control while sparing organs at risk.^9,10^ Retrospective comparisons demonstrated efficacy; patients who received SIB radiotherapy vs those who did not achieved superior 5-year OS (81.3% vs 58.3%) and PFS (75.0% vs 57.6%).^11^ Building on these findings, we initiated this phase 3 randomized clinical trial to definitively evaluate whether tumor bed SIB radiotherapy improves outcomes by 13% for patients with cervical cancer presenting with FT or OFT stromal invasion undergoing radical hysterectomy—a population currently lacking evidence-based therapeutic optimization.

## Methods

### Study Design and Participants

This phase 3 randomized clinical trial was designed to evaluate the superiority of concurrent SIB radiotherapy vs conventional CRT in terms of improving 3-year PFS. The trial protocol (Supplement 1) was approved by the ethics committee of Fudan University Shanghai Cancer Center. Written informed consent was obtained from all patients. This study followed the Consolidated Standards of Reporting Trials (CONSORT) reporting guidelines.

Patients with histologically confirmed FT or OFT stromal invasion according to institutional criteria were enrolled from October 15, 2019, to September 20, 2024. Patients with parametrial involvement were excluded from the study, as they routinely receive SIB radiotherapy at our institution due to the increased risk of local recurrence. Preoperative staging evaluations consisted of pelvic magnetic resonance imaging (MRI), positron emission tomography (PET) with computed tomography (CT), or contrast-enhanced chest-abdominal CT with pelvic MRI. All patients underwent radical hysterectomy with pelvic lymphadenectomy, with para-aortic lymphadenectomy selectively performed when preoperative imaging or intraoperative findings suggested nodal involvement.

### Randomization and Treatment

**Figure 1.**
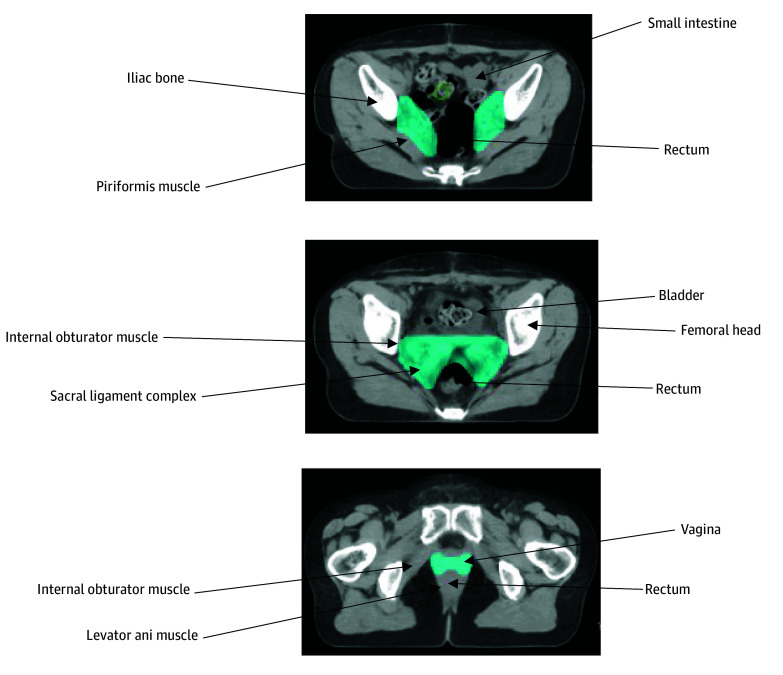
Cross-Sectional Images of Simultaneous Integrated Boost Radiotherapy Contours

Randomization was performed using computer-generated allocation, assigning patients equally to 2 groups of 233 each. The conventional CRT arm received pelvic intensity-modulated radiotherapy (IMRT) at 50.4 Gy delivered in 28 fractions with weekly cisplatin, 40 mg/m^2^. For patients with positive vaginal margins, a high dose-rate (HDR) intracavitary brachytherapy was performed using a vaginal ovoid applicator, with a prescription dose of 6 Gy per fraction (total 12 Gy in 2 fractions) delivered at a 0.5-cm depth beneath the vaginal mucosa. The experimental arm received an identical pelvic IMRT and cisplatin regimen, supplemented by SIB radiotherapy to the tumor bed (58.8 Gy/28 fractions). The SIB radiotherapy target volume extended 2 cm superior to the femoral head and 2 cm inferior from the top of the vaginal cuff (extended to 3 cm for positive margins in the SIB radiotherapy group) and included the bladder posterior wall anteriorly, the sacral ligament complex posteriorly, and the obturator internus and pubococcygeus muscles laterally (Figure 1). Both groups received extended-field radiotherapy for pathologically confirmed para-aortic lymph node involvement.

### Evaluation and Follow-Up

The primary end point was 3-year PFS, defined as the time from randomization to first radiologic or histopathologic progression or all-cause death. Secondary end points included 3-year OS, locoregional recurrence (pelvic region, vaginal cuff, or para-aortic nodes), distant metastasis (inguinal nodes, intra-abdominal sites, lungs, liver, or bones), tumor bed boost local control (absence of recurrence within the contoured SIB radiotherapy volume), and treatment-related toxic effects graded per the Common Terminology Criteria for Adverse Events, version 5.0.^12^

Follow-up adhered to Gynecologic Oncology Group guidelines^13^: quarterly evaluations for 2 years, biannual assessments through year 5, and annual visits thereafter. Standardized imaging included PET-CT or contrast-enhanced CT and pelvic MRI.

### Statistical Analysis

Statistical analysis was performed in October 2024. Based on institutional retrospective data, the estimated 3-year PFS for patients with 2018 FIGO (International Federation of Gynecology and Obstetrics) stage IB to IIA and IIIC cervical cancer receiving standard therapy is 65%.^11^ We hypothesized that the experimental intervention would increase 3-year PFS to 78%. With α = .05 and β = 0.1, accounting for a 5% attrition rate, and using a 1:1 randomization ratio, initial calculations yielded 211 patients per arm. To account for potential heterogeneity in treatment effects across studies, we inflated the sample size by 10%, resulting in a target of at least 233 patients per arm. This adjustment enhanced statistical power to approximately 95% while maintaining α = .05.

Continuous variables are expressed as median (SD) values and categorical variables as counts and percentages. Between-group comparisons used χ^2^ or Fisher exact tests. Survival analyses used Kaplan-Meier methodology with log-rank testing, complemented by multivariable Cox proportional hazards regression models to identify independent prognostic factors (hazard ratio [HR] with 95% CI). All tests were 2-tailed with α = .05, performed in SPSS, version 26.0 (IBM Corp).

## Results

### Clinical and Pathologic Characteristics

From October 15, 2019, to September 20, 2024, a total of 482 patients with pathologically confirmed FT or OFT stromal invasion after radical hysterectomy were screened for eligibility, and 466 patients were ultimately enrolled (eFigure 2 in Supplement 2). All outcomes were analyzed under the intention-to-treat principle. Baseline characteristics were balanced between the groups. The median age was 53 years (IQR, 46-59 years) in the non-SIB radiotherapy group vs 55 years (IQR, 48-60 years) in the SIB radiotherapy group (Table 1). Squamous cell carcinoma predominated in both groups (non-SIB radiotherapy group, 201 [86.3%]; SIB radiotherapy group, 190 [81.5%]), followed by adenocarcinoma (non–SIB radiotherapy group, 26 [11.2%]; SIB radiotherapy group, 35 [15.0%]) and adenosquamous carcinoma (non–SIB radiotherapy group, 6 [2.6%]; SIB radiotherapy group, 8 [3.4%]). Tumors 4 cm or more in size occurred in 106 patients in the non-SIB radiotherapy group (45.5%) vs 119 patients in the SIB radiotherapy group (51.1%), with lymphovascular space invasion (LVSI) present in 167 patients in the non-SIB radiotherapy group (71.7%) and 176 patients in the SIB radiotherapy group (75.5%). Rates of pelvic lymph node positivity (non–SIB radiotherapy group, 101 [43.3%]; SIB radiotherapy group, 115 [49.4%]), para-aortic node involvement (non–SIB radiotherapy group, 7 [3.0%]; SIB radiotherapy group, 2 [0.9%]), and positive vaginal margins (non–SIB radiotherapy group, 7 [3.0%]; SIB radiotherapy group, 3 [1.3%]) did not reach statistical significance.

**Table 1.** Patients’ Clinical and Pathologic Characteristics

Variable	Patients, No. (%)	
	Non-SIB radiotherapy (n = 233)	SIB radiotherapy (n = 233)
Age, median (IQR), y	53 (46-59)	55 (48-60)
Histologic type		
Squamous cell carcinoma	201 (86.3)	190 (81.5)
Adenocarcinoma	26 (11.2)	35 (15.0)
Adenosquamous carcinoma	6 (2.6)	8 (3.4)
FIGO stage (2018)		
IB1	9 (3.9)	7 (3.0)
IB2	8 (3.4)	11 (4.7)
IB3	5 (2.1)	7 (3.0)
IIA1	59 (25.3)	42 (18.0)
IIA2	51 (21.9)	51 (21.9)
IIIC1p	94 (40.3)	113 (48.5)
IIIC2p	7 (3.0)	2 (0.9)
Tumor size, cm		
<4	127 (54.5)	114 (48.9)
≥4	106 (45.5)	119 (51.1)
Perineural invasion		
Negative	173 (74.2)	173 (74.2)
Positive	60 (25.8)	60 (25.8)
Pelvic nodes		
Negative	132 (56.7)	118 (50.6)
Positive	101 (43.3)	115 (49.4)
Para-aortic nodes		
Negative	226 (97.0)	231 (99.1)
Positive	7 (3.0)	2 (0.9)
Surgical margin		
Negative	226 (97.0)	230 (98.7)
Positive	7 (3.0)	3 (1.3)
LVSI		
Negative	66 (28.3)	57 (24.5)
Positive	167 (71.7)	176 (75.5)
Conventional CRT (cisplatin)		
No	38 (16.3)	35 (15.0)
Yes	195 (83.7)	198 (85.0)

Abbreviations: CRT, chemoradiotherapy; FIGO, International Federation of Gynecology and Obstetrics; LVSI, lymphovascular space invasion; SIB, simultaneous integrated boost.

### Treatment Compliance

All patients completed prescribed radiotherapy: 50.4 Gy/28 fractions pelvic IMRT in both arms. In the non-SIB radiotherapy group, 7 patients with positive vaginal margins received supplemental HDR brachytherapy (12 Gy/2 fractions; EQD2, 65.56 Gy), and 7 with para-aortic node involvement underwent extended field irradiation. Concurrent cisplatin was administered to 195 patients in the non-SIB radiotherapy group (83.7%), with 38 patients (16.3%) excluded due to COVID-19 (Table 1).

The SIB radiotherapy group received additional tumor bed boosts of 58.8 Gy in 28 fractions. For margin-positive cases, this was combined with an extended external beam target volume (3 cm inferior from the top of the vaginal cuff) and a supplemental HDR brachytherapy fraction of 6 Gy/1 fraction, achieving a cumulative EQD2 of 67.29 Gy. Extended field radiotherapy was administered to 2 patient with positive para-aortic nodes. Concurrent cisplatin coverage reached 198 patients in the SIB radiotherapy group (85.0%), with 35 patients (15.0%) excluded for contraindications mirroring the non-SIB radiotherapy group (Table 1). No participants were lost to follow-up during the study period, and complete data were obtained for all primary and secondary end points without any missing values.

### Survival and Recurrence Outcomes

**Figure 2.**
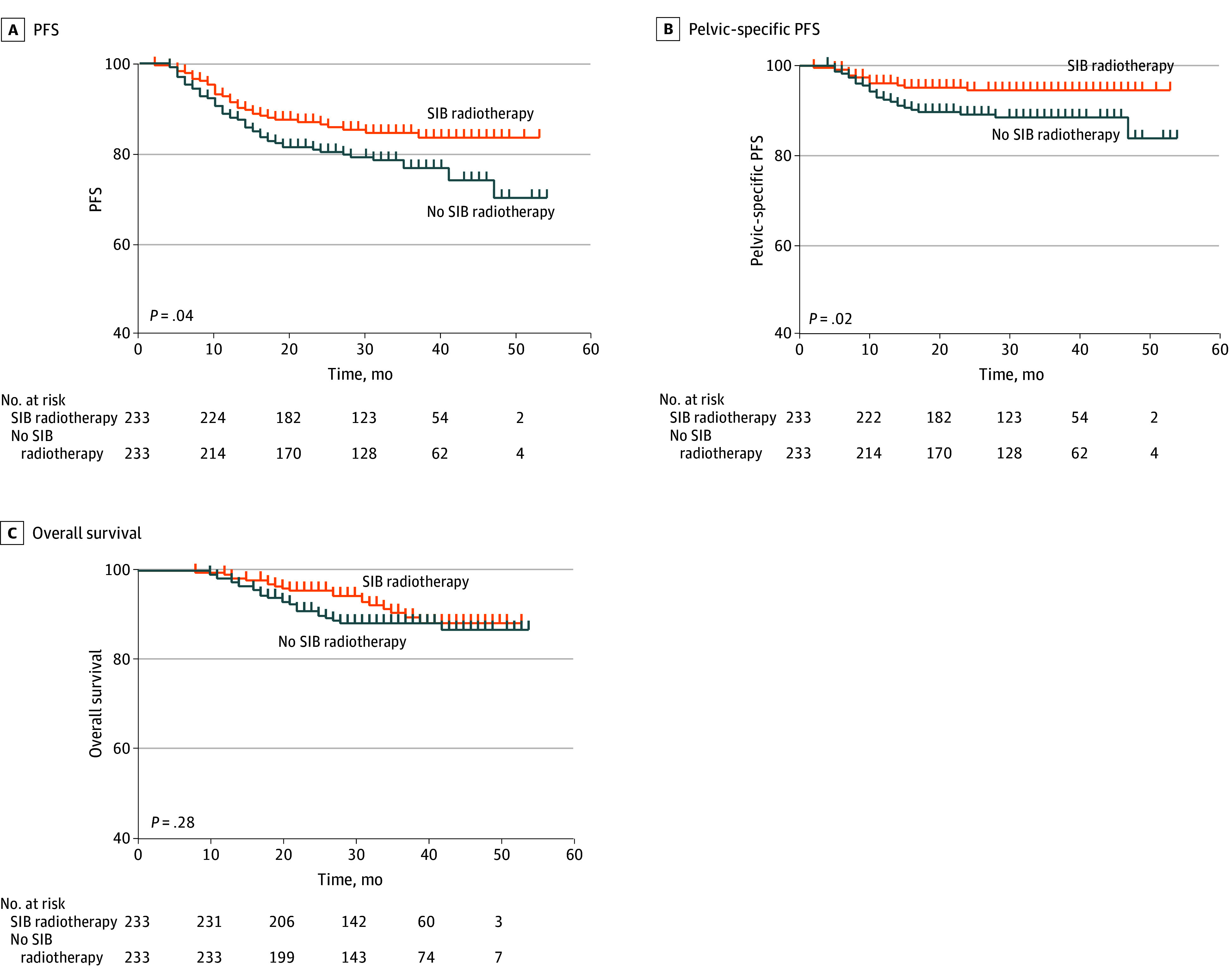
Survival Outcomes With or Without Simultaneous Integrated Boost (SIB) Radiotherapy Kaplan-Meier curves for progression-free survival (PFS) (A), pelvic-specific PFS (B), and overall survival (C).

At a median follow-up of 33 months (range, 8-54 months), survival analysis revealed significant 3-year PFS improvement with SIB radiotherapy (84.6% vs 76.8%; *P* = .04) (Figure 2A). Cox proportional hazards regression confirmed a significant treatment effect (HR, 0.64; 95% CI, 0.42-0.99; *P* = .04), corresponding to a 35.7% reduction in progression risk. Pelvic-specific PFS was higher in the SIB radiotherapy group (94.6% vs 88.5%; *P* = .02) (Figure 2B), as was tumor bed boost local control (97.6% vs 91.6%; *P* = .003) (eFigure 3A in Supplement 2). However, no significant differences emerged in 3-year OS (90.6% vs 88.3%; *P* = .28) (Figure 2C), distant metastasis-free survival (86.7% vs 82.4%; *P* = .33) (eFigure 3B in Supplement 2), or cancer-specific survival (91.7% vs 88.7%; *P* = .15) (eFigure 3C in Supplement 2).

The SIB radiotherapy group demonstrated superior local control. Isolated locoregional recurrence rates were decreased in the SIB radiotherapy group compared with the non-SIB radiotherapy group (5 [2.1%] vs 16 [6.9%]; *P* = .01). The incidence of concurrent locoregional recurrence and distant metastasis showed no statistically significant difference between the SIB and non-SIB groups (7 [3.0%] vs 11 [4.7%; *P* = .34) (Table 2). Likewise, the rates of isolated distant metastasis were comparable between the 2 groups (23 [9.9%] vs 26 [11.2%]; *P* = .65).

**Table 2.** Locoregional Recurrence and Distant Metastasis

Variable	Patients, No. (%)		*P* value
	Non-SIB radiotherapy (n = 233)	SIB radiotherapy (n = 233)	
Recurrent region			
None	180 (77.3)	198 (85.0)	.03
Locoregional recurrence	16 (6.9)	5 (2.1)	.01
Distant metastasis	26 (11.2)	23 (9.9)	.65
Both	11 (4.7)	7 (3.0)	.34

Abbreviation: SIB, simultaneous integrated boost.

### Prognostic Factor Analysis

**Figure 3.**
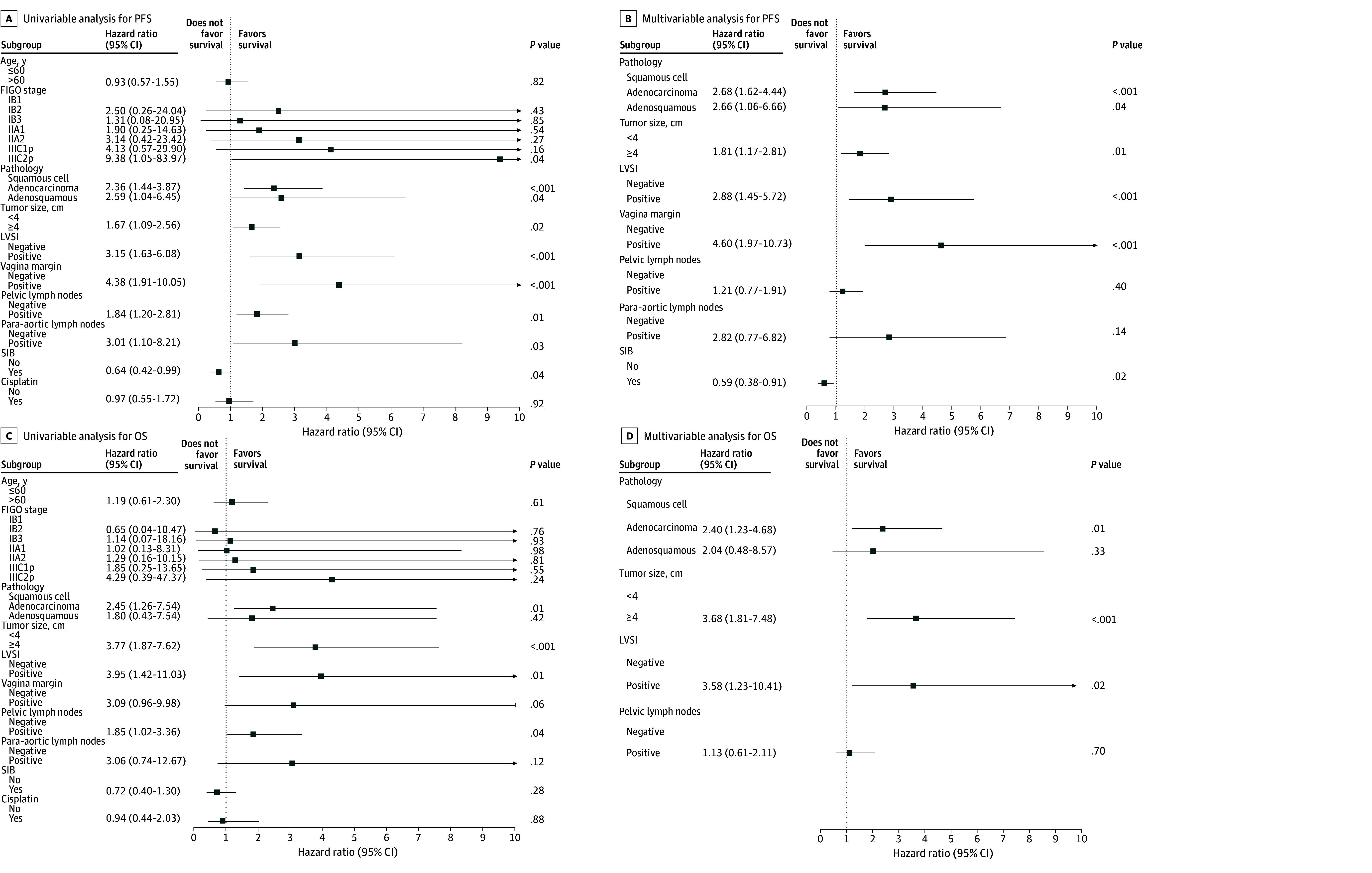
Risk Factors for Progression-Free Survival (PFS) and Overall Survival (OS) A, Univariable Cox proportional hazards regression analysis for PFS. B, Multivariable Cox proportional hazards regression analysis for PFS. C, Univariable Cox proportional hazards regression analysis for OS. D, Multivariable Cox proportional hazards regression analysis for OS. FIGO indicates International Federation of Gynecology and Obstetrics; LVSI, lymphovascular space invasion; and SIB, simultaneous integrated boost.

Multivariable Cox proportional hazards regression analysis, after addressing collinearity between FIGO IIIC2p and para-aortic nodal involvement, identified 3 independent risk factors for both PFS and OS: adenocarcinoma histology (HR for progression, 2.68; 95% CI, 1.62-4.44; HR for death, 2.40; 95% CI, 1.23-4.68), tumor size 4 cm or more (HR for progression, 1.81; 95% CI, 1.17-2.81; HR for death, 3.68; 95% CI, 1.81-7.48), and LVSI (HR for progression, 2.88; 95% CI, 1.45-5.72; HR for death, 3.58; 95% CI, 1.23-10.41) (Figure 3). SIB radiotherapy reduced recurrence risk by 41% (HR for progression, 0.59; 95% CI, 0.38-0.91), demonstrating consistent benefit across subgroups.

Histologic subtype exerted significant prognostic influence in PFS; compared with squamous carcinoma, adenocarcinoma doubled recurrent risk (HR, 2.68; 95% CI, 1.62-4.44) while adenosquamous carcinoma exhibited equivalent recurrence risk (HR, 2.66; 95% CI, 1.06-6.66). Positive vaginal margins conferred the most marked risk elevation, quadrupling recurrence probability (HR, 4.60; 95% CI, 1.97-10.73; Figure 3).

### Subgroup Analysis

Prespecified subgroup analyses revealed PFS benefits with SIB radiotherapy for patients with squamous cell carcinoma (HR, 0.57; 95% CI, 0.34-0.96; *P* = .03) and LVSI-positive patients (HR, 0.63; 95% CI, 0.40-0.98; *P* = .04), with absolute 3-year PFS improvements of 43% and 37%, respectively (eTable 2 in Supplement 2). No significant heterogeneity in treatment effect was observed across the subgroups analyzed (eTable 2 in Supplement 2).

### Postrelapse Survival Analysis

Postrelapse survival, defined as the time from confirmed recurrence (imaging, pathological, or clinical) to all-cause death, was analyzed for patients receiving salvage therapies. A total of 36 of 88 patients with relapse (40.9%) underwent immunotherapy or radiotherapy (including metastasis-directed and reirradiation strategies). Immunotherapy significantly prolonged postrelapse survival compared with nonimmunotherapy regimens (median postrelapse survival, 19 months [IQR, 12-26 months] vs 10 months [IQR, 7-18 months]; *P* = .001), with similar benefits observed for salvage radiotherapy (median postrelapse survival, 21.5 months [IQR, 10.8-25.5 months] vs 12 months [IQR, 7-19 months]; *P* = .03). Multivariable analysis confirmed both interventions as independent postrelapse survival risk factors—immunotherapy was associated with a 71% reduced risk of mortality (HR, 0.29; 95% CI, 0.13-0.65; *P* = .003), and postrelapse radiotherapy was associated with a 71% reduced risk of mortality (HR, 0.29; 95% CI, 0.09-0.94; *P* = .04), suggesting their potential synergistic prognostic value (eFigure 4 in Supplement 2).

### Toxic Effects and Safety Analyses

No treatment-related deaths occurred among the 466 patients. Hematologic toxic effects predominated during treatment, with grade 3 to 4 events occurring in 17 of 233 patients in the SIB radiotherapy group (7.3%) vs 12 of 233 patients in the non-SIB radiotherapy group (5.2%) (*P* = .13) (eTable 3 in Supplement 2). Gastrointestinal (nausea and vomiting) and genitourinary (urgency and tenesmus) symptoms were common but not statistically different between the 2 cohorts (eTable 4 in Supplement 2).

Critical complications included acute incomplete bowel obstruction (3 cases during the first week of radiotherapy, resolved conservatively) and late obstructions (9 cases: 5 in the SIB radiotherapy group vs 4 in the non-SIB radiotherapy group; *P* = .74), with 2 cases requiring enterostomy. Grade 3 limb edema occurred in 9 patients (3 in the SIB radiotherapy group vs 6 in the non-SIB radiotherapy group; *P* = .31), all managed nonsurgically. Profiles of toxic effects between arms showed no statistically significant divergence.

## Discussion

This phase 3 randomized clinical trial demonstrates that SIB radiotherapy (58.8 Gy/28 fractions) was associated with improved 3-year overall PFS and pelvic-specific PFS, corresponding to a one-third reduction in progression risk. The management of patients with cervical cancer presenting with FT or OFT stromal invasion after radical hysterectomy remains a critical unmet need in China, particularly for subgroups of patients with high-risk disease (stage IB3, IIA2, or IIIC). Conventional adjuvant radiotherapy often fails to eradicate microscopic residual disease in complex anatomical regions such as the bladder posterior wall and sacral ligaments. This localized efficacy attributable to precise dose escalation to subclinical lesions addresses a key limitation of standard therapy.

The absence of OS benefit should be interpreted cautiously given the nonrandomized postrelapse treatment allocation. Although a clinically meaningful 71% reduction in mortality risk was associated with both interventions, this represents an association rather than causal evidence of benefit. The populations receiving immunotherapy or salvage radiotherapy differed fundamentally from nonrecipients in disease biology and recurrence patterns. Specifically, radiotherapy candidates inherently had localized recurrences with a better prognosis rather than disseminated disease, while immunotherapy recipients likely had fewer comorbidities and better performance status.

The integration of immune checkpoint inhibitors and advanced radiotherapy after recurrence has redefined recurrence management. Our institutional experience aligns with KEYNOTE-826 and CheckMate358 outcomes,^14,15^ in which immune checkpoint inhibitors improved prognosis in recurrent disease. Pembrolizumab combined with CRT recently demonstrated survival benefits in locally advanced disease,^16^ while the CALLA study revealed pronounced PFS improvements specifically for patients with high programmed death-ligand 1 expression (tumor area positivity, ≥20%).^17^

Nonsquamous histologic subtypes adenocarcinoma and adenosquamous carcinoma demonstrated significantly worse outcomes compared with squamous cell carcinoma, with approximately doubled risks of both disease recurrence and cancer-related death in our study. Adenocarcinoma exhibits an inferior response to radiotherapy and a higher propensity for metastases,^18,19,20,21^ evidenced by a 44% recurrence rate in observational studies vs 28% for squamous cell carcinoma.^22^ Meanwhile, radiotherapy reduces recurrence more dramatically in adenocarcinoma (from 44% to 9%) than squamous cell carcinoma (from 28% to 20%),^22^ suggesting that conventional Sedlis criteria risk stratification inadequately addresses adenocarcinoma’s unique radiosensitivity profile.

The prognostic significance of tumor factors exhibits histologic specificity; while stromal invasion depth dominates in squamous cell carcinoma, a tumor size of 4 cm or more emerges as the cardinal risk factor in adenocarcinoma,^23^ conferring 80% higher recurrence risk and 3.7-fold mortality risk in our cohort. The 2018 FIGO staging validation studies corroborate this dimensional threshold, revealing survival gradients of 97% for IB1, 92.1% for IB2, and 83.1% for IB3 in 5-year OS.^24^ This dimensional continuum extends to stage II disease, in which tumors larger than 4 cm independently estimate reduced 5-year OS and elevated metastasis risk.^25^ Mechanistically, larger tumors demonstrate higher rates of lymph node metastasis, local recurrence, and distant metastasis.^26^

Lymphovascular space invasion demonstrates robust correlations with advanced tumor stage, nodal metastasis, and the anatomical distribution of positive lymph nodes,^27,28,29^ with meta-analyses confirming the association between LVSI, pelvic lymph node metastasis, and parametrial invasion in cervical cancer.^30^ In our cohort, LVSI positivity tripled recurrence risk and increased mortality risk 3.6-fold, retaining significance in multivariable-adjusted models. These findings position LVSI as a critical factor for metastatic propensity, surpassing conventional staging in prognostic stratification.

For patients with cervical cancer presenting with positive surgical margins, NCCN guidelines advocate external beam radiotherapy with concurrent platinum-based chemotherapy, supplemented by brachytherapy to optimize local control. Supporting this approach, combined external beam radiotherapy and brachytherapy demonstrates superior 3-year OS vs external beam radiotherapy alone.^31^ In our cohort, both therapeutic strategies achieved biologically effective doses greater than 70 Gy. Consistent with the validation by Lee et al^32^ of external beam boost efficacy, no significant outcome difference emerged between groups (*P* = .78). However, multivariable analysis identified positive vaginal margins as a potent independent risk factors of recurrence (HR, 4.60; 95% CI, 1.97-10.73), with distant metastases predominating in the SIB radiotherapy group. These findings highlight the necessity for systemic therapeutic intensification beyond localized dose escalation in margin-positive disease.

The FIGO 2018 staging system redefined lymph node metastasis as stage IIIC, reflecting its prognostic significance. Lymph node involvement correlates strongly with deep stromal invasion tumors larger than 2 cm, LVSI, and parametrial extension factors independently associated with nodal metastasis.^33^ The GOG 0263 study did not demonstrate a statistically significant survival advantage for conventional CRT compared with RT alone in intermediate-risk patient cohorts.^34^ However, Peters et al^35^ subsequently established the superiority of conventional CRT in treating patients with high-risk disease characteristics through their analysis. Our multivariable analysis indicated that stromal invasion depth, tumor size, and LVSI supersede nodal status as stronger prognostic factors in this cohort. The independent prognostic significance of nodal involvement did not retain statistical significance in this model. A similar pattern was observed among patients treated with conventional CRT.^36^

Although the experimental regimen significantly improved 3-year PFS, the observed 7.8% absolute gain fell short of the hypothesized 13% improvement. This attenuated effect likely reflects multiple factors, including higher-than-expected control group performance (76.8% vs a historical baseline of 65%), suggesting that contemporary advances in standard care diminished the measurable benefit or inherent biological heterogeneity despite balanced baselines. The 95% CI width (0.42-0.99) reflects precision limitations from fewer events but confirms therapeutic superiority. To better characterize treatment effects, we could extend the follow-up period or pursue multicenter replication.

### Limitations

This study has some limitations. Our findings reflect a surgery-first paradigm predominant in China (especially for select cases of IB3, IIA2, or IIIC disease), where SIB radiotherapy target volumes were optimized for postsurgical recurrence patterns. These patterns may differ from recurrence landscapes in nonsurgical cohorts treated with definitive CRT, potentially limiting generalizability to CRT-dominant regions. Pathologic classifications of stromal invasion depth followed institutional protocols, which may lack global harmonization. Although acute toxic effects were comparable, late effects remain uncertain given immature long-term data. SIB radiotherapy–related late toxic effect risks may escalate over time, necessitating extended surveillance. Ongoing 5-year follow-up will clarify late survival trends and toxic effects, which will provide valuable insights into therapeutic efficacy and late-onset adverse events, guiding future clinical applications.

## Conclusions

In this randomized clinical trial of patients with cervical cancer presenting with FT or OFT stromal invasion after radical surgery, SIB radiotherapy established itself as an effective strategy for improving PFS and locoregional control. The demonstrated clinical benefits, coupled with comparable profiles of toxic effects, strongly support integrating SIB radiotherapy into standard adjuvant therapy protocols for patients in this cohort.

## Notes

Supplement 1.
Trial Protocol

Supplement 2. **eTable 1.** Detailed Description of the Depth of Stromal Invasion of Cervical Cancer in our Institution **eTable 2.** Subgroup Analysis and Interaction Tests of Treatment Effect on PFS **eTable 3.** Hematological Toxicity Reactions During Treatment **eTable 4.** Adverse Effects on the Gastrointestinal and Urinary Systems **eFigure 1.** Diagram of the Depth of Stromal Invasion of Cervical Cancer in our Institution **eFigure 2.** Patient Flowchart **eFigure 3.** (A) Kaplan-Meier Curves for TBBLC; (B) Kaplan-Meier Curves for DMFS; (C) Kaplan-Meier Curves for CSS **eFigure 4.** (A) Kaplan-Meier Curves for PRS With Immunotherapy; (B) Kaplan-Meier Curves for PRS With Radiotherapy; (C) Multivariable Cox Regression Analysis for PRS

Supplement 3.
Data Sharing Statement
