# Supplementary material for: Chemoradiotherapy With or Without Simultaneous Integrated Boost for Cervical Cancer With Full-Thickness Stromal Invasion: A Phase 3 Randomized Clinical Trial
Source: JAMA Netw Open. 2025 Sep 19;8(9):e2532501. doi: 10.1001/jamanetworkopen.2025.32501 (PMC12449725; doi:10.1001/jamanetworkopen.2025.32501)
Supplement: Supplement 1. — Trial Protocol [file jamanetwopen-e2532501-s001.pdf]

Phase III Randomized Controlled Clinical Study on Adjuvant Chemoradiotherapy with or Without Simultaneous Integrated Boost via External Beam Radiation Therapy for Patients with Full-Thickness and Outer Full-Thickness Stromal Invasion Following Radical Surgery for Early-Stage Cervical Cancer

(Protocol Version: 2019-GO-002, July 20, 2019)

I. Study Background

Cervical cancer is currently the third most common malignancy and the fourth leading cause of cancer-related death among women worldwide, posing a significant threat to women's health [1]. Globally, there are approximately 529,000 new cases and 275,000 deaths annually [2]。Over 80% of cervical cancer cases occur in developing countries. In China, there are approximately 130,000 new cases and 20,000 deaths annually, with recent trends indicating regional increases in incidence and earlier onset of the disease [3-5]。

The optimal treatment strategy for cervical cancer depends on factors such as patient age, tumor stage, pathological type, fertility requirements, comorbidities, and institutional resources. Therefore, a comprehensive evaluation and multidisciplinary team discussion are essential to formulate individualized treatment plans, including surgery, radiotherapy, or chemoradiotherapy.

For early-stage cervical cancer (Stage I–IIA2), radical surgery and radical radiotherapy yield comparable outcomes, with similar 5-year survival, mortality, and complication rates. For bulky tumors (e.g., IB3 or IIA2), the NCCN guidelines prioritize radical radiotherapy (Category 2B recommendation for radical surgery), while in China, treatment decisions are tailored based on institutional surgical expertise and radiotherapy capabilities. Postoperative adjuvant concurrent chemoradiotherapy is recommended for patients with high-risk recurrence factors (parametrial invasion, positive vaginal margins, or lymph node metastasis). For intermediate-risk factors (tumor size, lymphovascular space invasion [LVSI], depth of

stromal invasion, based on Sedlis criteria), adjuvant radiotherapy ± chemotherapy is advised to reduce pelvic recurrence and improve survival. The Gynecologic Oncology Group (GOG) has established indications for postoperative radiotherapy:

Lymphovascular Space Invasion	Cervical Stroma Invasion	Tumor Size (cm)
+	Deep 1/3	Any size
+	Middle 1/3	≥ 2
+	Superficial 1/3	≥ 5
-	Middle or Deep 1/3	≥ 4

If any of the above criteria are met, postoperative adjuvant radiotherapy is recommended. The radiotherapy field should minimally include the vaginal stump 3-4 cm downward, parametrial tissue, and adjacent lymph node beds (e.g., external iliac and internal iliac lymph nodes). If common Iliac lymph nodes and paraaortic lymph nodes metastasis is confirmed, the upper boundary of the radiation field should be extended accordingly. Standard fractionation radiotherapy is typically recommended, with a dose of 45-50 Gy. For non-resectable enlarged lymph nodes, a dose of 10-15 Gy should be added using highly conformal external beam radiotherapy (EBRT). When using high-dose EBRT, particular attention should be paid to the radiation tolerance doses of normal tissues. External beam radiotherapy boost should be administered to areas where residual tumor may remain after surgery, to a total dose of approximately 60 Gy. For rare histological types such as adenocarcinoma, carcinosarcoma, and neuroendocrine carcinoma, which are less sensitive to radiotherapy, an appropriate increase in dose is recommended.

Previous studies have indicated that patients with adenocarcinoma of the cervix who undergo surgery followed by radiotherapy have a worse prognosis compared to those with squamous cell carcinoma or adenosquamous carcinoma, suggesting that adenocarcinoma is an independent poor prognostic factor. Moreover, patients with adenocarcinoma and lymph node metastasis have an even worse prognosis [4]. Therefore, a "four-factor" model theory for adjuvant radiotherapy in cervical adenocarcinoma has been proposed, which includes: tumor diameter >3 cm, LVSI (+),

extracervical 1/3 stromal invasion, and histological type of adenocarcinoma. Guidelines recommend that adjuvant radiotherapy is beneficial for patient survival prognosis if any two of the above factors are present.

However, for patients with full-thickness or outer full-thickness stromal invasion after radical surgery, the current adjuvant radiotherapy approach is the same as for other patients with intermediate or high-risk factors. Deep stromal invasion of cervical cancer has already been identified as an intermediate-risk factor for recurrence in cervical cancer. Previous retrospective studies have also found that the depth of cervical tumor invasion is an important prognostic indicator for patients with cervical cancer. Several studies have reported a significant correlation between deep stromal invasion and pelvic lymph node metastasis [5-6]. Research using three-dimensional pathological assessment has shown that approximately 30-60% of clinically "early" tumors (Stage IB-IIA) in patients with cervical cancer exhibit subclinical or occult parametrial spread, which is significantly associated with pelvic lymph node metastasis [7-10]。However, previous studies have been limited to deep stromal invasion, and there are currently no clinical or pathological reports that consider full-thickness or greater involvement of cervical tumors as a high-risk factor for recurrence. A retrospective analysis of the clinical and pathological data of patients with early-stage cervical cancer after radical surgery found that cervical stromal invasion is an independent prognostic factor for 5-year overall survival (OS) and disease-free survival (DFS) [11]。In our clinical practice, we have observed that many patients who underwent radical surgery for cervical cancer have pathological findings indicating tumor involvement of the full thickness or greater of the cervix. Review of their preoperative imaging studies consistently shows varying degrees of tumor invasion into surrounding tissues. Therefore, we believe that these patients should be distinguished from those with only deep stromal invasion but not reaching the full thickness or greater during postoperative adjuvant chemoradiotherapy.。

During external beam radiotherapy, the cervical lesion is generally not given an additional boost, and boosting only the lymph nodes is sufficient. Moreover, most

cervical squamous cell carcinomas are sensitive to radiotherapy, and tumors typically regress by approximately 50-75% during the mid-to-late stages of radiotherapy, at which point brachytherapy is added to achieve radical treatment for cervical cancer. However, for patients who have undergone radical surgery, the surgical margin is approximately 2-3 cm from the vaginal stump. If parametrial positivity is indicated, brachytherapy may not reach an effective distance. Therefore, for these patients who may have residual tumor, the conventional external beam radiotherapy dose of 45-50.4 Gy may not achieve a radical dose for the tumor. Hence, it is necessary to administer a simultaneous integrated boost to the tumor bed during external beam radiotherapy to 60 Gy.

Approximately 29-38% of patients with early-stage cervical cancer experience recurrence after surgery, and recurrent cervical cancer is challenging to treat, with a low 5-year survival rate [12]. Few studies have investigated the factors associated with recurrence in cervical cancer. Relevant literature reports that 75% of cervical cancer patients experience recurrence within 2 years of initial treatment, and the recurrence rate after treatment for invasive cervical cancer is 29%, with 35% of recurrences localized to the pelvis [13]. Currently, treatment for localized pelvic recurrences primarily involves surgery or radiotherapy. Due to the close proximity of the vaginal stump to the posterior wall of the bladder, postoperative pelvic adhesions, and vaginal wall fibrosis caused by postoperative radiotherapy, repeat surgery after recurrence is very difficult. The NCCN guidelines recommend a treatment plan of radiotherapy combined with chemotherapy for patients with recurrent cervical cancer [14]。Pelvic external beam radiotherapy and brachytherapy are crucial for reducing the risk of vaginal stump recurrence and improving overall survival in patients with Stage I-II cervical cancer after radical surgery. Patients with high-risk and intermediate-risk factors after surgery should be actively given adjuvant radiotherapy.

This study aims to evaluate the potential benefits of adjuvant chemoradiotherapy with a simultaneous integrated boost for patients with full-thickness or outer full-thickness cervical stromal invasion after radical surgery for

early-stage cervical cancer. By analyzing the survival outcomes of patients after treatment, this study seeks to assess and explore the clinical relationship between full-thickness or outer full-thickness stromal invasion and recurrence and survival prognosis, as well as the guiding significance of adjuvant therapy for such patients.

参考文献

[1] Ferlay J, Shin HR, Bray F, Forman D, Mathers C, Parkin DM. Estimates of worldwide burden of cancer in 2008: GLOBOCAN 2008. Int J Cancer 2010; 127(12): 2893-917.

[2] Jemal A，Bray F，Center MM，Ferlay J，Ward E，Forman D. Global cancer statistics. CA Cancer J Clin 2011; 61:1-22.

[3] Wang SS, Sherman ME, Hildesheim A, Lacey Jr JV, Devesa S. Cervical adenocarcinoma and squamous cell carcinoma incidence trends among white women and black women in the United States for 1976-2000. Cancer 2004; 100: 1035-44.

[4] Sherman ME, Wang SS, Carreon J，Devesa SS. Mortality trends for cervical squamous and adenocarcinoma in the United States. Cancer 2005; 103(6): 1258-64.

[5] Bray F, Carstensen B, Moiler H, Zappa M, Zakelj MP, Lawrence G, et al. Incidence trends of adenocarcinoma of the cervix in 13 European countries. Cancer Epidemiol Biomark Prev 2005; 14: 2191-9.

[6] Nanthamongkolkul K, Hanprasertpong J. Predictive Factors of Pelvic Lymph Node Metastasis in Early-Stage Cervical Cancer. Oncol Res Treat. 2018;41(4):194-198.doi: 10.1159/000485840.

[7] Benedetti-Panici P, Maneschi F, D'Andrea G, Cutillo G, Rabitti C, Congiu M, Coronetta F, Capelli A: Early cervical carcinoma: the natural history of lymph node involvement redefined on the basis of thorough parametrectomy and giant section study. Cancer 2000;88:2267-2274.

[8] Hanprasertpong J, Jiamset I, Geater A, Leetanaporn K, Peerawong T: Impact of time interval between radical hysterectomy with pelvic node dissection and initial adjuvant therapy on oncological outcomes of early stage cervical cancer. J Gynecol

Oncol 2017;28:e42

[9] Klapdor R, Hertel H, Soergel P, Jentschke M, Hillemanns P: Application of sentinel lymph node dissection in gynecological cancers: results of a survey among German hospitals. Arch Gynecol Obstet 2017;295:713-720.

[10] Kadkhodayan S, Hasanzadeh M, Treglia G, Azad A, Yousefi Z, Zarifmahmoudi L, Sadeghi R: Sentinel node biopsy for lymph nodal staging of uterine cervix cancer: a systematic review and meta-analysis of the pertinent literature. Eur J Surg Oncol 2015;41:1-20.

[11] 张子规,赵虎,李文，等.淋巴脉管间隙浸润对早期宫颈癌预后的影响分析[J].中国实用妇科与产科杂志,2018,34(8):907-912.

[12] Zhou H. Clinical analysis of concurrent radiotherapy and chemotherapy for advanced or recurrent cervical cancer. Chin Fore Med Res, 2015, 13(11) : 43-44.

[13] Kasamatsu T, Onda T, Yamada T, et a1. Clinical aspects and prognosis of pelvic recurrence of cervical carcinoma. Int J Gynaecol Obstet, 2005, 89(1): 39. 44.

[14] Metindir J, Bilir G. Prognostic factors affecting disease-free survival in early-stage cervical cancer patients undergoing radical hysterectomy and pelvic-para-aortic lymphadenectomy. Eur J Gynaecol Oncol, 2007, 28(1): 28-32.

II. Research Content and Technical Key Points

2.1 Research Objectives:

This study aims to evaluate the potential benefits of adjuvant chemoradiotherapy with or without a simultaneous integrated boost in patients with early-stage cervical cancer who have undergone radical surgery and have full-thickness or outer full-thickness stromal invasion.

- **Primary Objective**: 3-year progression-free survival.
- **Secondary Objectives**: 3-year overall survival rate, acute and late toxicities, and progression patterns.

  Progression-free survival (PFS): PFS is defined as time from randomization to first radiological/histopathological progression or all-cause death.

  Overall survival (OS): Refers to the time period from the date of receiving study treatment to the date of death (any cause). Subjects without an observed death will be censored at the time of confirmed survival.

  Acute and late toxicities: KPS score, vital signs, Physical examination, laboratory tests (blood routine examination, routine urine and stool test), comprehensive metabolic panel, coagulation test and so on), ECG, Cardiac ultrasound, adverse events (AEs), AEs is recorded based on CTCAE 5.0 criteria.

  Progression patterns: Analyze the progression pathways of the disease, including local-regional recurrence (pelvic region/vaginal cuff/para-aortic nodes), distant metastasis (inguinal nodes, intra-abdominal sites, lungs, liver, bones), tumor bed boost local control (absence of recurrence within the contoured SIB volume)

2.2 Innovativeness:

This is the first randomized controlled Phase III clinical trial to investigate adjuvant chemoradiotherapy with or without a simultaneous integrated boost in patients with early-stage cervical cancer who have undergone radical surgery and have full-thickness or outer full-thickness stromal invasion.

2.2.1 Study Design:

This is a single-center, randomized, Phase III trial.

- **Group A**: Standard adjuvant chemoradiotherapy + simultaneous integrated boost.
- **Group B**: Standard adjuvant chemoradiotherapy without simultaneous integrated boost.

All patients in both groups will receive standard pelvic external beam radiotherapy. Concurrent chemotherapy with cisplatin will be administered at a dose of 40 mg/m^2 weekly for 5 weeks.

2.2.2 Study Population:

Patients who underwent radical surgery for cervical cancer at our center between October 2019 and October 2020, with postoperative pathology indicating full-thickness or outer full-thickness stromal invasion.

2.2.3 Randomization:

Patients will be randomized using a minimization method into two groups: standard adjuvant chemoradiotherapy with simultaneous integrated boost or standard adjuvant chemoradiotherapy without simultaneous integrated boost. Randomization will be performed using a random number table, with a 1:1 allocation ratio.

2.3.1 Inclusion Criteria:

1. Pathological confirmation of full-thickness or outer full-thickness stromal invasion after radical surgery for cervical cancer.
2. Age 18-65 years.
3. ECOG performance status 0-2.
4. Histological type: squamous cell carcinoma, adenocarcinoma, or adenosquamous carcinoma of the cervix.
5. WBC $\geq 3.5 \times 10^9/L$, ANC $\geq 2.0 \times 10^9/L$, platelets $\geq 100 \times 10^9/L$.
6. Bilirubin $\leq 1.5 \times$ ULN, AST or ALT $\leq 2.5 \times$ ULN.
7. Good renal function.
8. Signed informed consent.

2.3.2 Exclusion Criteria:

1. Postoperative pathology indicating parametrial involvement.
2. Patients who received chemotherapy before radiotherapy.
3. Bilateral hydronephrosis, inability to place ureteral stents, or inability to perform nephrostomy.
4. Distant metastasis.
5. History of ulcerative colitis or Crohn's disease.
6. Other active malignancies.
7. Pregnant or breastfeeding patients.
8. Contraindications to cisplatin chemotherapy.
9. Severe underlying medical conditions that preclude safe administration of study treatment, including but not limited to persistent or active infection, symptomatic congestive heart failure, unstable angina, arrhythmias, or psychiatric/social conditions that may limit adherence to study requirements.
10. HIV positive.

2.3.3 Withdrawal Criteria:

Participation in this study is voluntary, and patients may withdraw at any time. Patients may be withdrawn or excluded from study treatment for the following reasons:

1. Inability to continue follow-up.
2. Patient withdrawal of consent.
3. Changes in the patient's condition that the investigator deems necessitate stopping treatment.

2.4 Trial Process

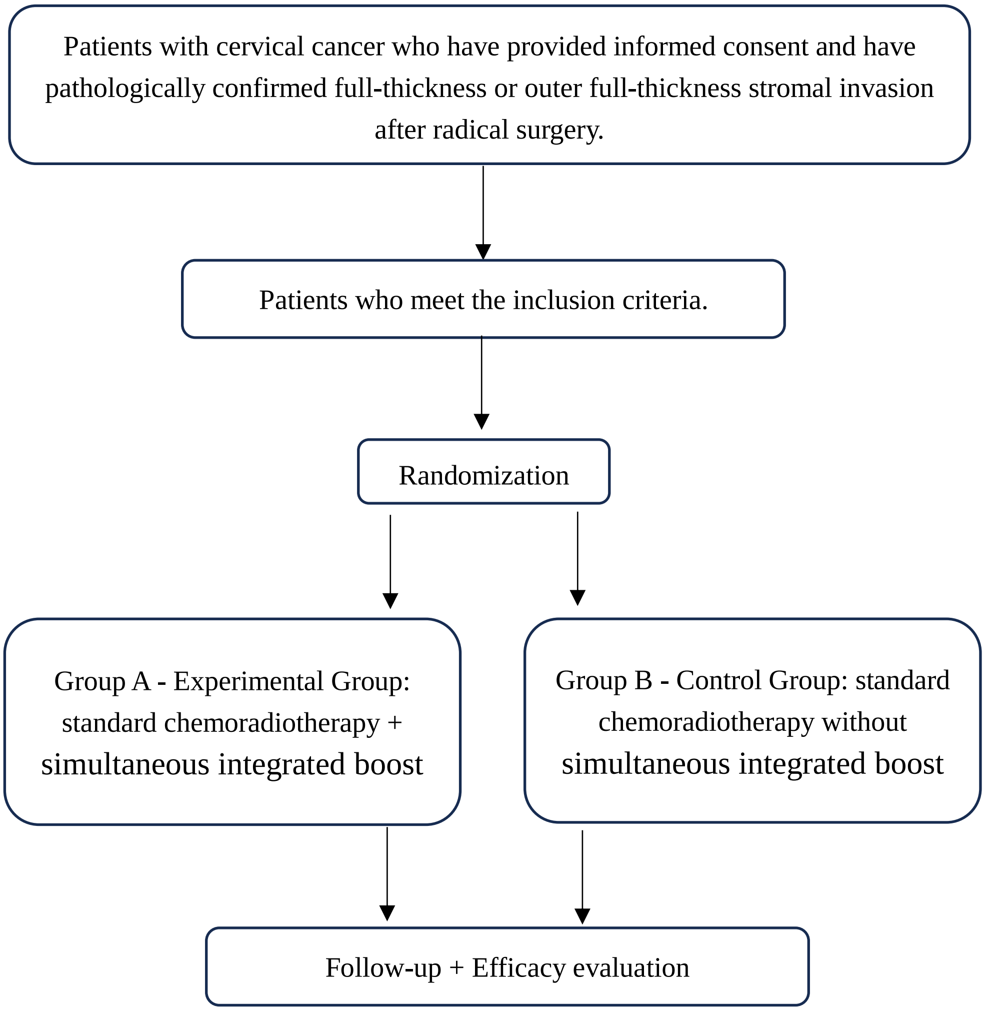

2.5 Main Research Content:

2.5.1 Radiotherapy

Radiotherapy must begin within 4 weeks after randomization.

2.5.1.1 External Beam Radiotherapy Equipment:

Linear accelerator, 6MV-X-rays.

2.5.1.2 External Beam Radiotherapy Technique:

- Patients are required to drink 500 ml of water one hour before simulation to ensure bladder fullness.
- CT simulation is performed with the upper boundary at the inferior edge of

T10 and the lower boundary at the vulvar opening, with 5 mm slice thickness.

- Radiotherapy is delivered using intensity-modulated radiation therapy (IMRT) techniques.
- The pelvic field CTV includes internal iliac, external iliac, common iliac, obturator, presacral, uterine cervix, corpus uteri, adnexal areas, parametrial tissue, part or all of the vagina, and the extended field CTV includes the pelvic field plus the para-aortic lymph node drainage area, with the upper boundary at the level of the left renal vein.
- Specific target delineation refers to the "Fudan University Shanghai Cancer Center Gynecological Oncology Target Delineation Guidelines."
- The PTV is expanded 0.8 cm from the CTV.
- Patients are required to drink 500 ml of water one hour before radiotherapy to maintain bladder fullness.

The upper boundary of the external beam plan is generally at the level of the aortic bifurcation, and the lower boundary is determined based on the extent of vaginal involvement. The target area must include the tumor bed, parametrium, uterosacral ligaments, presacral lymph nodes, and other potentially involved lymph nodes, as well as sufficient vaginal tissue. If common iliac or para-aortic lymph node metastasis has occurred, extended field irradiation is required, with the upper boundary reaching the level of the renal vessels or extended cephalad based on the lymph node involvement.

2.5.1.3 External Beam Dose:

Conventional fractionation is used, with 1.8 Gy per fraction, 5 fractions per week. Total dose: 50.4 Gy over 5 weeks. The dose uniformity within the target area should be within ±5%, while evaluating organs at risk, such as the rectum, sigmoid colon, bladder, small intestine, ilium, sacrococcygeal bone, pubis, femoral head, and femoral neck.

**PTV**	V50.4 >= 95%, Dmax<= 107%
**PTV-boost**	V58.8 >= 95%, Dmax<= 107%,

**Rectum**	V60<=30%; D2cc<=105%
**Bladder**	V60<=30%; D2cc <=105%
**Small Intestine**	V30<=40%; V40<=30%; D2cc<=105%
**Femoral Head**	V30<20%
**Kidneys**	V15<50%
**Spinal Cord**	Dmax<45Gy

2.5.1.4 Simultaneous Integrated Boost:

During the external beam target delineation process, the tumor bed area will be given a simultaneous integrated boost of 58.80 Gy/2 8 fractions. The upper boundary is 2 cm above the femoral head, the lower boundary is 2cm inferior to the vaginal cuff, the two laterally sides are the medial edge of the internal obturator muscle and the pubococcygeus muscle, the front boundary includes the posterior wall of the bladder, and the posteriorly boundary is the part where the residual sacral ligament and the rectal mesentery fuse with the sacral ligament.

2.5.2 Chemotherapy:

Concurrent chemotherapy with cisplatin will be administered during radiotherapy. The specific dose is 40 mg/m^2, administered based on patient weight on the day of radiotherapy. Chemotherapy will not be given on days without radiotherapy, and a maximum of five doses will be administered. Chemotherapy should be scheduled from Monday to Wednesday, avoiding Thursday and Friday. All toxicities will be graded according to the National Cancer Institute (NCI) Common Terminology Criteria for Adverse Events (CTCAE) version 4.0. No chemotherapy will be administered until all drug-related toxicities (except anemia) of $\geq$ Grade 2 have resolved to Grade 1. It is recommended to correct anemia (Hb < 10 g/dL) with blood transfusion as soon as possible. No dose escalation will be performed in this study. No chemotherapy will be administered during radiotherapy delays. Radiotherapy will not be delayed due to chemotherapy-related toxicities unless the investigator deems the patient too weak to undergo treatment. Only one dose reduction is allowed for

cisplatin administration.

Drug	Starting Dose Level	Dose Level -1
**Cisplatin**	40mg/m^2	30mg/m^2

Drug-Related Toxicity Adjustments:

Toxicity	Parameter	Adjustment
**Febrile Neutropenia/ANC**	First occurrence of febrile neutropenia (≥ Grade 3) or ANC < 0.5 × 10^9/L for >7 days	Hold for 1 week. Repeat blood tests. If ANC resolves to Grade 1, resume at reduced dose level. If ANC does not resolve, discontinue cisplatin.
**Febrile Neutropenia/ANC**	Second occurrence	Discontinue cisplatin.
ANC	Non-complex (no fever/infection) ANC < 0.5 × 10^9/L for <7 days	No dose reduction, but do not retreat until ANC resolves to Grade 1.
**Platelets**	First occurrence of Grade 4 thrombocytopenia or bleeding with Grade 3 thrombocytopenia	Hold for 1 week. Repeat blood tests. If platelets >75 × 10^9/L, resume at reduced dose level. If thrombocytopenia does not resolve, discontinue cisplatin.
**Platelets**	Second occurrence	Discontinue cisplatin.
**Platelets**	Grade 3 – non-complex (no bleeding)	Hold treatment until platelets >75 × 10^9/L, then resume at current dose level (no adjustment).
**Nausea/Vomiting**	Grade 3 or 4	Hold treatment until resolution to Grade 1 (with supportive care). Resume at reduced dose level.
**Nausea/Vomiting**	Second occurrence	Discontinue cisplatin

**Serum Creatinine/GFR**	GFR < 50 ml/min (Cockcroft-Gault) or GFR < 40 ml/min (measured creatinine or EDTA clearance)	Hold for 1 week. Repeat blood tests. If GFR recovers to the above level at the same dose, resume treatment. If not recovered after 1 week, discontinue cisplatin.
**Neurotoxicity – Peripheral Neuropathy**	≥ Grade 2	Hold treatment until neuropathy resolves to Grade 1. Resume at reduced dose level. If neuropathy does not resolve within 21 days, discontinue cisplatin.
**Tinnitus or Hearing Loss**	Grade 3 or 4	Discontinue cisplatin.
**Tinnitus or Hearing Loss**	Grade 2	Hold treatment until resolution to Grade 1, then resume at reduced dose level. Audiometry should be performed to determine if hearing loss is reversible.
**Fatigue**	Any grade	No dose reduction required.
**Other Non-Hematologic Toxicities**	≥ Grade 3	Hold treatment until toxicity resolves to Grade 1, then resume at the same dose. If toxicity does not resolve to Grade 1 within 21 days, discontinue cisplatin.

2.5.3 Other Medications:

Anti-emetic therapy: Aprepitant 125 mg, dexamethasone 8 mg, ondansetron 8 mg; or a standard combination of a corticosteroid and a 5HT antagonist.

2.5.4 Efficacy Evaluation:

All patients will undergo routine baseline assessments, including clinical review, routine blood tests, and imaging studies (CT, MRI, or PET scans). Weekly follow-ups will be conducted during chemoradiotherapy, and clinical examinations will be performed every 3 weeks during adjuvant chemotherapy. Patients will then be

observed every 3 months for 2 years, followed by every 6 months for 5 years. It is planned that all patients will complete the treatment study, except in cases of disease progression or toxicity preventing further treatment. Regardless of treatment completion, quarterly follow-ups will be conducted for 2 years, then every 6 months for 3 years or until death. After completing all study treatments, treatment efficacy will be determined 6 months after randomization based on clinical examination and chest/abdominal/pelvic CT results. If suspicious lesions are present, PET scans should be repeated 4 to 6 months after completion of chemoradiotherapy, with priority given to PET-CT scans. CT scans should also be performed upon any recurrence to determine the observed pattern of recurrence. Progression-free survival (PFS) is defined as the time from randomization to investigator-determined disease progression or death from any cause. Overall survival is defined as the time from randomization to death from any cause.

2.5.5 Safety Evaluation:

Adverse events (AEs) are defined as any unfavorable medical event occurring in a patient or clinical study subject during the course of radiotherapy and chemotherapy. AEs do not necessarily have a causal relationship with the treatment. AEs include any unfavorable or unintended sign (including abnormal laboratory findings), symptom, or disease temporally associated with the study drug administration, regardless of whether it is considered treatment-related.

AEs include the following:

- All suspected drug or device reactions.
- All reactions from drugs or devices, including overdoses, drug abuse, drug sensitivity, toxicity, or failure of expected pharmacological effects (if applicable).
- Apparently unrelated illnesses, including exacerbations of pre-existing conditions (severity, frequency).
- Injuries or accidents.
- Abnormalities in physiological tests or physical examinations requiring

clinical intervention or further investigation (beyond predefined repeat tests).

- Laboratory abnormalities requiring clinical intervention or further investigation (beyond predefined laboratory tests).
- Any unfavorable event occurring during the reporting period specified in the protocol that the investigator considers related to the drug or device.

Even if they do not meet the criteria for serious adverse events (SAEs), AEs must be reported as AEs. SAEs are defined as any unfavorable medical event occurring at any dose that:

- Results in death.
- Is life-threatening (the subject is at immediate risk of dying at the time of the event).
- Requires inpatient hospitalization or prolongation of existing hospitalization.
- Results in persistent or significant disability or incapacity.
- Results in congenital anomaly/birth defect.
- Is judged by the investigator to be a serious or protocol-defined important medical event requiring intervention.

2.5.6 Sample Size and Feasibility Analysis:

Based on the retrospective analysis data of our center, the estimated 3-year progression-free survival (PFS) for patients with FIGO stage IB, IIA, and IIIC cervical cancer following primary treatment was observed to be 65%. This investigation is designed to demonstrate a statistically significant 13% improvement in PFS, corresponding to an anticipated 3-year PFS rate of 65% in the control cohort compared to 78% in the experimental arm. Recruitment is expected to take 2 years, with a 3-year follow-up period. Utilizing a conventional significance level (α) of 0.05 and statistical power ($1 - \beta$) of 0.9 ($\beta = 0.1$), with a 5% anticipated attrition rate and 1:1 randomization scheme, sample size calculations determined a requirement of 211 cases per group. This determination was cross-validated through PASS 15, nQuery, and G*Power statistical software.

Considering substantial variability in PFS rates reported across existing literature,

we implemented a 10% sample size augmentation strategy to enhance detection sensitivity for clinically meaningful differences, mitigate type II error risks, and improve result validity. This adjustment results in a revised minimum enrollment target of 233 participants per group, yielding an anticipated statistical power of approximately 95% for detecting the hypothesized treatment effect.

This study plans to enroll at least 466 patients with cervical cancer who have undergone radical surgery and have full-thickness or outer full-thickness stromal invasion. Fudan University Shanghai Cancer Center treats nearly 1,000 cases of cervical malignancies annually, including approximately 300 cases of radical radiotherapy and 600 cases of postoperative adjuvant radiotherapy. Thus, the hospital has sufficient capacity to complete the required number of cases for this trial. The Gynecological Oncology Department has been conducting intensity-modulated radiation therapy (IMRT) for cervical cancer since 2007 and has completed approximately 4,000 cases of IMRT for cervical cancer, demonstrating technical maturity. Additionally, the hospital's molecular pathology laboratory has mature detection techniques.

2.5.7 Statistical Analysis:

2.5.7.1 Data Management

All data collected during the trial will be recorded on case report forms (CRFs) and then entered into a secure, password - protected electronic database.

Data will be checked for accuracy and completeness, and any discrepancies or missing data will be queried and resolved in a timely manner.

Regular data monitoring and cleaning procedures will be conducted throughout the study to ensure data quality.

2.5.7.2 Statistical Analysis Plan

The statistical analysis plan (SAP) will be developed prior to database lock and will specify all analysis methods, including the primary and secondary outcome measures, subgroup analyses, and handling of missing data.

The SAP will be followed strictly during the analysis phase to avoid data - driven

analyses and ensure the integrity of the study results.

2.5.7.3 Analysis Population

The primary analysis will be conducted on the intention - to - treat (ITT) population, which includes all randomly assigned patients, regardless of their actual treatment received or whether they completed the study as planned.

For safety analyses, all patients who received at least one dose of the study treatment will be included in the safety population.

2.5.7.4 Sample Size Calculation and Rationale:

Refer to section 2.5.6.

2.5.7.5 Description of Statistical Methods:

All enrolled patients were randomized into groups using computer-generated allocation.

The primary outcome will be compared between groups.

2.5.7.5.1 Descriptive Statistics

For continuous variables, summary statistics such as mean, standard deviation, median, and interquartile range will be reported.

For categorical variables, frequency counts and percentages will be presented.

Appropriate graphical displays (e.g., histograms, box plots, bar charts) will be used to visualize the distribution of key variables.

2.5.7.5.2 Comparative Analyses

The primary outcome will be compared between groups using independent - samples t - test for continuous variables assuming normal distribution, Mann - Whitney U test for non - normally distributed continuous variables, chi - square test or Fisher's exact test for categorical variables.

For time - to - event outcomes, Kaplan - Meier survival curves will be estimated, and the log - rank test will be used to compare groups. Hazard ratios and their 95%

confidence intervals will also be calculated using Cox proportional hazards regression models.

For secondary outcomes, similar comparative methods will be applied as appropriate for each outcome type.

2.5.7.6 Handling of Missing Data

The amount and pattern of missing data will be assessed for each key outcome variable.

Sensitivity analyses will be conducted to evaluate the potential impact of missing data on the study results.

2.5.7.7 Subgroup Analyses

Specified subgroup analyses will be conducted to explore the treatment effect in different subgroups of patients, including but not limited to SIB or Non-SIB groups, tumor size groups, etc.

These subgroup analyses will be interpreted with caution, considering the potential for multiple comparisons and the exploratory nature of these analyses.

2.5.7.8 Adverse Event Analysis

The incidence and types of adverse events (AEs) will be summarized for each treatment group.

The severity and relatedness of AEs to the study intervention will also be evaluated and reported.

Comparative analyses of safety data between groups will be performed using appropriate statistical methods (chi - square tests for categorical AE data).

2.5.7.9 Statistical Software

All statistical analyses will be performed using SPSS 19.

2.5.7.10 Reporting of Results

Results will be reported following the CONSORT guidelines for randomized controlled trials.

Both statistical significance and clinical relevance will be considered when interpreting the findings.

All P - values presented are two - sided, and P - values less than 0.05 will be considered statistically significant.

Confidence intervals will be reported at the 95% level for key outcome measures to quantify the precision of the estimates.
