# Supplementary material for: Chemoradiotherapy With or Without Simultaneous Integrated Boost for Cervical Cancer With Full-Thickness Stromal Invasion: A Phase 3 Randomized Clinical Trial
Source: JAMA Netw Open. 2025 Sep 19;8(9):e2532501. doi: 10.1001/jamanetworkopen.2025.32501 (PMC12449725; doi:10.1001/jamanetworkopen.2025.32501)
Supplement: Supplement 2. — eTable 1. Detailed Description of the Depth of Stromal Invasion of Cervical Cancer in our Institution eTable 2. Subgroup Analysis and Interaction Tests of Treatment Effect on PFS eTable 3. Hematological Toxicity Reactions During Treatment eTable 4. Adverse Effects on the Gastrointestinal and Urinary Systems eFigure 1. Diagram of the Depth of Stromal Invasion of Cervical Cancer in our Institution eFigure 2. Patient Flowchart eFigure 3. (A) Kaplan-Meier Curves for TBBLC; (B) Kaplan-Meier Curves for DMFS; (C) Kaplan-Meier Curves for CSS eFigure 4. (A) Kaplan-Meier Curves for PRS With Immunotherapy; (B) Kaplan-Meier Curves for PRS With Radiotherapy; (C) Multivariable Cox Regression Analysis for PRS [file jamanetwopen-e2532501-s002.pdf]

Supplementary Online Content

Liu S, Fang C, Wu X, Zhu J, Ke G. Chemoradiotherapy with or without simultaneous integrated boost for cervical cancer with full-thickness stromal invasion: a phase 3 randomized clinical trial. *JAMA Netw Open.* 2025;8(9):e2532501. doi:10.1001/jamanetworkopen.2025.32501

eTable 1. Detailed Description of the Depth of Stromal Invasion of Cervical Cancer in our Institution
eTable 2. Subgroup Analysis and Interaction Tests of Treatment Effect on PFS
eTable 3. Hematological Toxicity Reactions During Treatment
eTable 4. Adverse Effects on the Gastrointestinal and Urinary Systems
eFigure 1. Diagram of the Depth of Stromal Invasion of Cervical Cancer in our Institution
eFigure 2. Patient Flowchart
eFigure 3. (A) Kaplan-Meier Curves for TBBLC; (B) Kaplan-Meier Curves for DMFS; (C) Kaplan-Meier Curves for CSS
eFigure 4. (A) Kaplan-Meier Curves for PRS With Immunotherapy; (B) Kaplan-Meier Curves for PRS With Radiotherapy; (C) Multivariable Cox Regression Analysis for PRS

This supplementary material has been provided by the authors to give readers additional information about their work.

eTable 1. Detailed Description of the Depth of Stromal Invasion of Cervical Cancer in our Institution

Depth of stromal invasion	Description
Superficial	Tumor invasion limited to the upper 1/3 of the cervical stroma
Middle	Tumor invasion extending beyond the upper 1/3 but not reaching the deep 1/3 of the cervical stroma
Deep	Tumor invasion extending into the lower 1/3 of the cervical stroma without complete stromal involvement
Full-thickness	Complete stromal involvement without parametrial transition zone extension
Outer full-thickness	Microscopic transition zone involvement without macroscopic parametrial spread

eTable 2. Subgroup Analysis and Interaction Tests of Treatment Effect on PFS

Subgroup Characteristic	Subgroup Category	No	Hazard Ratio (95% CI)	P Value	P interaction
Overall		466	0.64(0.42-0.99)	0.04	
Histology	Squamous	391	0.57（0.34-0.96）	0.03	0.71
	Adenocarcinoma	61	0.60（0.25-1.39）	0.23	
	Adenosquamous	14	1.33（0.22-7.59）	0.76	
Tumor Size	Tumor size ＜ 4cm	193	0.49（0.20-1.18）	0.11	0.51
	Tumor size≥4cm	273	0.68（0.41-1.10）	0.12	
LVSI	Negative	123	0.53（0.14-2.05）	0.36	0.79
	Positive	343	0.63（0.40-0.98）	0.04	
LN Status	Negative	242	0.53（0.26-1.09）	0.09	0.58
	Positive	224	0.69（0.40-1.17）	0.17	

© 2025 Liu S et al. *JAMA Network Open.*

eTable 3. Hematological Toxicity Reactions During Treatment

**Group**	**No Toxicity**	**G1-2**	**G3**	**G4**	**P value**
SIB Group	22（4.7%）	194（41.6%）	16（3.4%）	1（0.2%）	0.127
Non-SIB Group	26（5.6%）	195（41.8%）	11（2.4%）	1（0.2%）	
Total	10.3%	83.4%	5.8%	0.4%	

© 2025 Liu S et al. *JAMA Network Open.*

eTable 4. Adverse Effects on the Gastrointestinal and Urinary Systems

**Adverse effects**	**G1-2**		**G3-4**		**P value**
	**SIB**	**Non-SIB**	**SIB**	**Non-SIB**	
Gastrointestinal reactions					
Acute toxic reactions	162（69.5%）	137（58.8%）	2（0.9%）	1（0.4%）	0.068
Late toxic reactions	34（14.6%）	23（9.9%）	1（0.4%）	1（0.4%）	0.486
Urinary system reactions					
Acute toxic reactions	121（51.9%）	102（43.8%）	3（1.3%）	2（0.9%）	0.286
Late toxic reactions	46（19.7%）	35（15.1%）	1（0.4%）	0（0.0%）	0.329

© 2025 Liu S et al. *JAMA Network Open.*

eFigure 1. Diagram of the Depth of Stromal Invasion of Cervical Cancer in our Institution

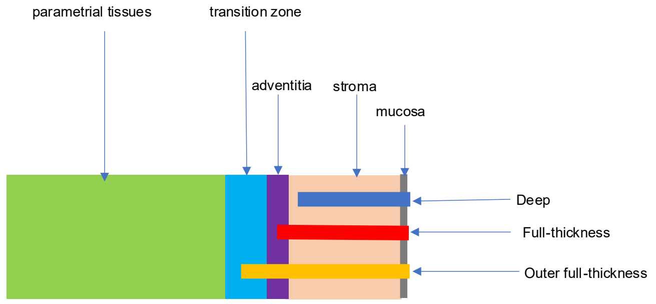

© 2025 Liu S et al. *JAMA Network Open.*

eFigure 2. Patient Flowchart

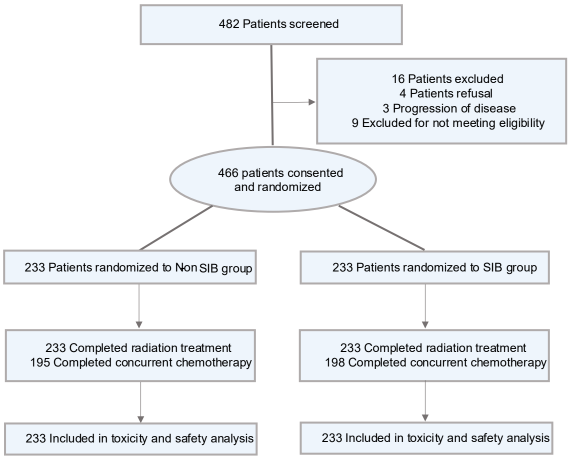

© 2025 Liu S et al. *JAMA Network Open.*

eFigure 3. (A) Kaplan-Meier Curves for TBBLC; (B) Kaplan-Meier Curves for DMFS; (C) Kaplan-Meier Curves for CSS

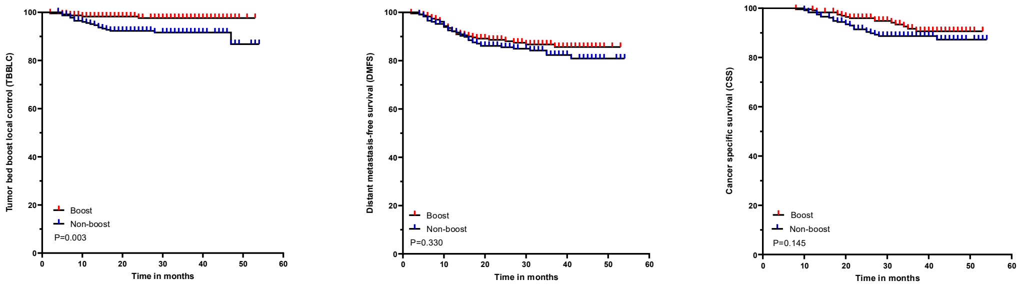

Number at risk: n（%）

	0	10	20	30	40	50
Boost	233	222	182	123	54	2
Non-boost	233	214	170	128	62	4

A

Number at risk: n（%）

	0	10	20	30	40	50
Boost	233	222	182	123	54	2
Non-boost	233	214	170	128	62	4

B

Number at risk: n（%）

	0	10	20	30	40	50
Boost	233	232	206	142	60	3
Non-boost	233	233	199	143	74	7

C

© 2025 Liu S et al. *JAMA Network Open.*

eFigure 4. (A) Kaplan-Meier Curves for PRS With Immunotherapy; (B) Kaplan-Meier Curves for PRS With Radiotherapy; (C) Multivariable Cox Regression Analysis for PRS

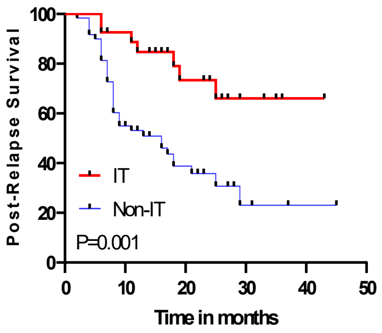

A

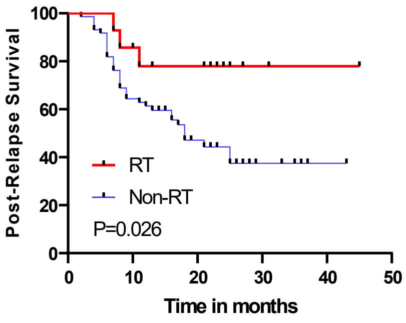

B

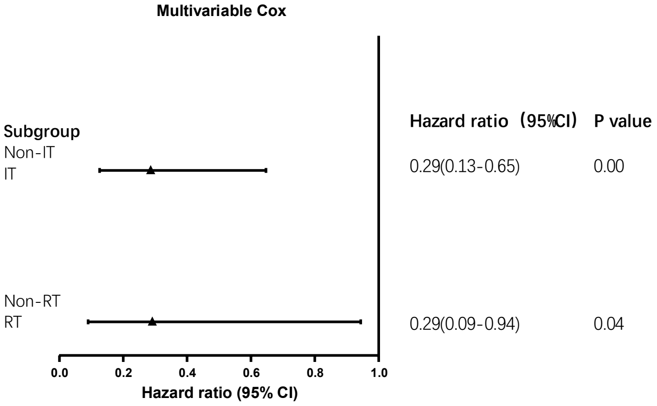

C

© 2025 Liu S et al. *JAMA Network Open.*
