# Supplementary material for: Chemoradiotherapy With or Without Simultaneous Integrated Boost for Cervical Cancer With Full-Thickness Stromal Invasion: A Phase 3 Randomized Clinical Trial
Source: JAMA Netw Open. 2025 Sep 19;8(9):e2532501. doi: 10.1001/jamanetworkopen.2025.32501 (PMC12449725; doi:10.1001/jamanetworkopen.2025.32501)
Supplement: Supplement 3. — Data Sharing Statement [file jamanetwopen-e2532501-s003.pdf]

Data Sharing Statement

Liu. Chemoradiotherapy With or Without Simultaneous Integrated Boost for Cervical Cancer With Full-Thickness Stromal Invasion. *JAMA Netw Open*. Published September 19, 2025. doi:10.1001/jamanetworkopen.2025.32501

Data
Additional Information: https://www.chictr.org.cn/showproj.html?proj=45487
Data available: Yes
Data types: Other (please specify)
Additional Information: The data and materials that support the findings of this study are provided within the manuscript and available from the corresponding author upon reasonable request.
How to access data: kegh5734@126.com
When available: With publication

Supporting Documents
Document types: Statistical/analytic code
How to access documents: kegh5734@126.com
When available: With publication

Additional Information
Who can access the data: Researchers whose proposed use of the data has been approved
Types of analyses: for a specified purpose
Mechanisms of data availability: after approval of a proposal
